# Surfactant assisted exfoliation of near infrared fluorescent silicate nanosheets†

**DOI:** 10.1039/d3ra04083f

**Published:** 2023-07-11

**Authors:** Bjoern Hill, Smitha Abraham, Anas Akhtar, Gabriele Selvaggio, Kristina Tschulik, Sebastian Kruss

**Affiliations:** a Department of Chemistry, Ruhr Universität Bochum 44801 Bochum Germany Sebastian.Kruss@rub.de; b Analytical Chemistry II, Ruhr Universität Bochum 44801 Bochum; c Fraunhofer Institute for Microelectronic Circuits and Systems 47057 Duisburg Germany; d Center for Nanointegration Duisburg-Essen (CENIDE) 47057 Duisburg Germany

## Abstract

Fluorophores that emit light in the near infrared (NIR) are advantageous in photonics and imaging due to minimal light scattering, absorption, phototoxicity and autofluorescence in this spectral region. The layered silicate Egyptian blue (CaCuSi_4_O_10_) emits as a bulk material bright and stable fluorescence in the NIR and is a promising NIR fluorescent material for (bio)photonics. Here, we demonstrate a surfactant-based (mild) exfoliation procedure to produce nanosheets (EB-NS) of high monodispersity, heights down to 1 nm and diameters <20 nm in large quantities. The approach combines planetary ball milling, surfactant assisted bath sonication and centrifugation steps. It avoids the impurities that are typical for the harsh conditions of tip-sonication. Several solvents and surfactants were tested and we found the highest yield for sodium dodecyl benzyl sulfate (SDBS) and water. The NIR fluorescence emission (*λ*_em_ ≈ 930–940 nm) is not affected by this procedure, is extremely stable and is not affected by quenchers. This enables the use of EB-NS for macroscopic patterning/barcoding of materials in the NIR. In summary, we present a simple and mild route to NIR fluorescent nanosheets that promise high potential as NIR fluorophores for optical applications.

## Abstract 2

Egyptian blue (CaCuSi_4_O_10_) is exfoliated into 2D nanosheets of high monodispersity down to monolayers by a mild surfactant-assisted procedure. These nanosheets are fluorescent in the near infrared (NIR) and useful for (bio)photonics.

## Graphical abstract

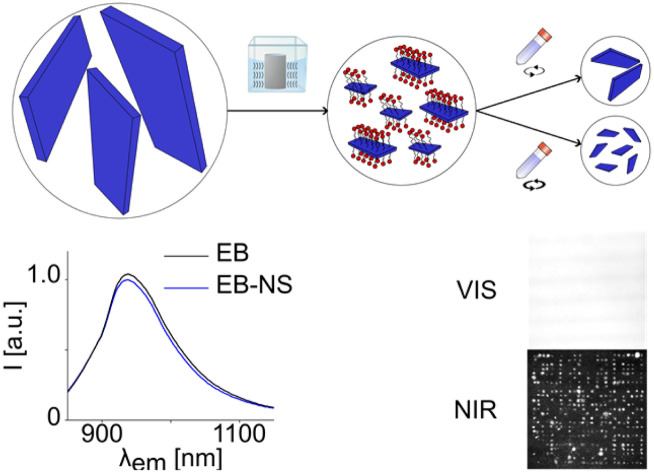

## Introduction

Fluorescent materials are important building blocks in many applications. For example, they can be used as labels or sensors for biological samples. In particular, near-infrared (NIR) fluorescence offers several advantages for the study of biological systems.^1,2^ Many biomolecules show autofluorescence, which can interfere with a specific fluorescence signature. In the NIR, this autofluorescence is significantly reduced. Absorption and scattering of radiation in the NIR are also significantly lower compared to visible light.^2^ Therefore, signals in the NIR have higher tissue penetration, resulting in better signal-to-noise ratios and substantially lower phototoxicity.^1^ Organic NIR fluorescent dyes such as cyanines^3,4^ and modified boron dipyrromethenes (BODIPYs)^5,6^ have been successfully applied for a number of biological studies, but they may have disadvantages such as fast bleaching, low quantum yield or biocompatibility. Another option is NIR fluorescent nanomaterials, such as semiconducting single-walled carbon nanotubes (SWCNTs),^7–11^ quantum dots,^12^ or doped nanoparticles.^13^

Two-dimensional (2D) nanomaterials provide electronic and photophysical properties that are of interest for a broad range of applications and technologies, for example in photonics, electronics, catalysis, and particularly in biomedical applications.^14–16^ The most prominent representative of this class is graphene,^17,18^ among other 2D materials such as transition metal dichalcogenides (TMDs)^19,20^ or silicate clays.^21^ Two routes are available for generating 2D nanomaterials. On the one hand, there are bottom-up synthesis processes for 2D materials, such as chemical vapor deposition (CVD), epitaxial growth or wet chemical processes.^22,23^ These can be used to produce thin, large-area and uniform layers, but only in small quantities and at high cost. The other way is top-down processes like exfoliation or delamination. Liquid phase exfoliation (LPE) provides a scalable and easy way to produce 2D nanomaterials from bulk material, which can be easily deposited from the liquid phase onto different surfaces.^24,25^ LPE has been optimized for several established 2D nanomaterials for various applications.^14,26^

A promising material for biophotonics that displays fluorescence in the NIR and the potential to be exfoliated into 2D nanosheets due to its layered structure is Egyptian blue (CaCuSi_4_O_10_, EB).^27–30^ EB is the oldest artificial pigment known to mankind being used as early as 2500 BC in ancient Egypt to decorate various artworks.^31,32^ In modern times, EB appears of great interest in particular as a novel NIR fluorophore. EB has a tetragonal crystal structure consisting of parallel layers of silicate tetrahedra weakly bound by calcium ions.^33–36^ Within the silicate framework are copper ions (Cu^2+^), which are the reason for the photophysical properties of this material. The NIR fluorescence of EB is attributed to the ^2^B_2g_–^2^B_1g_ electronic transition of the copper ion.^37,38^ Bulk EB emits fluorescence with a maximum at about 930 nm, and a broad excitation spectrum in the visible wavelength range, from 550–700 nm.^37,39^ This fluorescence shows remarkable stability. Furthermore, EB fluorescence is characterized by a very high quantum yield (up to 11%) compared to typical NIR fluorescent dyes and a fluorescence lifetime (100–150 μs) longer than the vast majority of NIR fluorescent materials.^38,40,41^

Due to the layered structure of the calcium–copper silicate possessing weak inter-layer bonds, it is possible to exfoliate EB in Egyptian blue nanosheets (EB-NS). By simple long-term stirring in hot water, it is possible to obtain sheets with dimensions in the nano-range.^29^ Some exfoliation techniques were explored, mostly consisting of a milling step and a sonication step.^29,30,39^ Among the approaches reported so far in literature, a combination of planetary ball milling and tip sonication, each in aqueous dispersion, resulted particularly successful in producing EB-NS down to monolayer thickness and with improved monodispersity.^40^ Besides, a first approach of exfoliation by sonication in surfactants was also demonstrated but did not focus on monodisperse samples in the monolayer range.^42^ It was shown that the characteristic photophysical properties, such as the wavelength and intensity of fluorescence, were preserved upon exfoliation to EB-NS.^30^ Besides exfoliation, a bottom-up synthesis of large platelets of EB from a synthetic layered sodium silicate has been reported.^43^

The biocompatibility of EB-NS has been demonstrated by cell viability assays and *in vivo* studies.^30,40^ Due to its high brightness, the NIR fluorescence of EB-NS can be imaged even using standard Si-based cameras, which are less sensitive for NIR signals.^40^ By combining the properties of bright NIR fluorescence and the ability to exfoliate it into nanosheets, EB-NS offer great potential in the biomedical field for bioimaging, sensing, tissue engineering, or even photothermal therapy. Functionalization of EB-NS has been demonstrated and used for targeting of cells.^44^ The remarkable stability of the EB fluorescence provides furthermore opportunities as reference material in ratiometric sensors.^45^ Other types of applications such as ink,^29^ fingerprint dusting powder,^42,46^ scaffolds for selective enrichment of phosphopeptides^47^ luminescent solar concentrators,^48^ or as composite for wound healing or bone reconstruction^49^ were also proposed.

Although the exfoliation of EB to EB-NS has been demonstrated, there are not yet EB-NS samples of high monodispersity and with layer thicknesses in the range of a few- or monolayers. For other 2D materials, a variety of exfoliation techniques have been explored. For graphene, for example, decades of research have led to a catalog of procedures.^17,50,51^ In particular, liquid phase exfoliation has been optimized for these materials by screening a wide range of different exfoliation media like different (organic) solvents or surfactants.^14,24,51^

Here, we conduct a screening of different exfoliation methods and media for EB-NS. This enables us to produce EB-NS with high monodispersity and sizes down to the nm-level. We report the photophysical properties of the obtained EB-NS and present possible applications.

## Results and discussion

Both the physical and chemical properties and the interaction with the environment of a nanomaterial are affected by its size. This applies as well to the use of these materials as nanosensors or fluorescent labels. Particularly when using them as tool in biological samples, it is advantageous if these are as small as possible so that they cannot influence the biological system itself. EB-NS can be applied in biophotonics, for example for fluorescence imaging of organisms down to the cellular level.^30,45^ For this reason, it is of major interest to find ways to produce nanosheets as small and as monodisperse as possible. In the process of EB-NS preparation, the exfoliation to 2D materials with preferably small layer thickness plays an important role, however also the lateral range has to be considered and should be reduced down to the nanometer scale.

To obtain EB-NS with defined height and lateral size, we developed an exfoliation process for EB. To find a path to smaller and monodisperse nanosheets, we performed a screening of different exfoliation methods and surfactants and characterized the obtained nanomaterials.

### Exfoliation of EB into EB-NS

A combination of PB milling and tip sonication in water was found to be the most efficient method to obtain small EB-NS so far,^27^ thus it was chosen as starting point for further investigation. We therefore performed a general exfoliation protocol consisting of two steps: first, a planetary ball (PB) milling step was carried out followed by either tip sonication or bath sonication. For particle size selection, liquid phase centrifugation was used.

The first step of this protocol was PB milling in water. Grinding techniques such as PB milling are commonly used comminute various minerals, in particular silicates down to the μm range and below.^52,53^ PB milling is an essential part of the exfoliation process. If PB milling is omitted and the sample is only sonicated, this leads to a drastic decrease in the yield to nanoparticles (Fig. S1b†), but also to an increase in the minimum size of the smallest nanoparticles produced. For this reason, all further experiments were performed with milled samples.

The slurry obtained by milling was then centrifuged to remove larger particles. The cut-off size was chosen to be around 1 μm, so that only particles with a hydrodynamic radius in the nanometer range were retained in the sample. After milling, the sample contained particles in the range of 100 nm to few μm with a reasonable yield of sub-μm particles, as confirmed by using dynamic light scattering (DLS) measurements as an approximate measure for size (Fig. S1a†).

For the second central step of the exfoliation process, two different exfoliation methods were compared: tip sonication and bath sonication. Tip sonication is a widely used method for nanomaterial dispersion, for example of carbon nanotubes.^7^ Its application in the exfoliation of EB-NS has already been successfully demonstrated.^30,40^ Bath sonication has already been successfully applied for the exfoliation of other nanomaterials.^26,51^

**Fig. 1:**
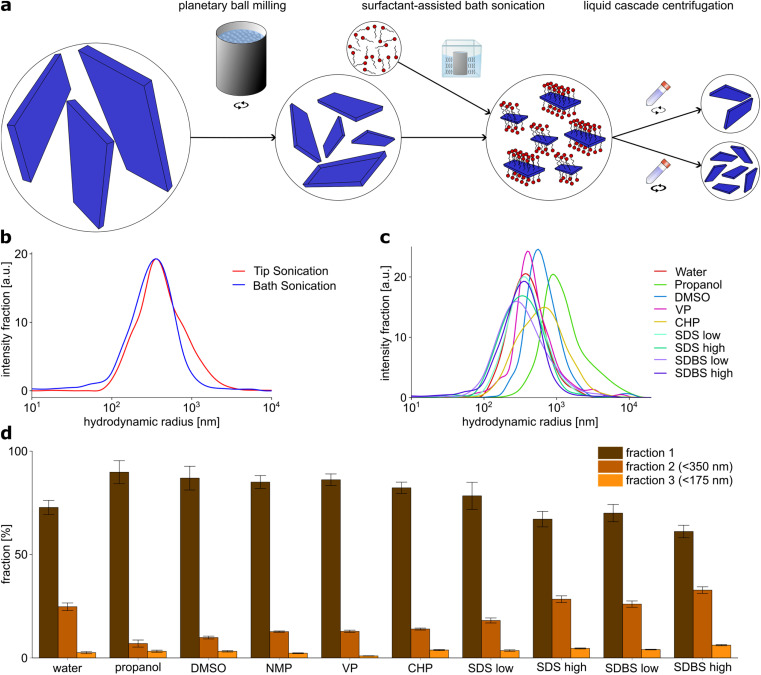
Liquid phase exfoliation of EB-NS assisted by surfactants. (a) Schematic of the exfoliation procedure. Bulk powder EB is exfoliated into EB-NS *via* a two-step exfoliation procedure. First, EB is grinded in water using a planetary ball mill to reduce the particle size. Liquid phase centrifugation is then performed for size selection and removal of larger particles. As the second step, ultrasonic treatment of the sample, either as tip sonication or bath sonication is conducted with different surfactants and solvents. Further centrifugation steps are carried out as liquid cascade centrifugation to receive EB-NS in different particle size fractions. Note that the surfactants are still present if they have not been removed but they are not shown for the final product for clarity. (b) Size distribution of EB-NS exfoliated in water by different sonication methods, measured by DLS. For each sample the particle size distribution is given as the intensity fraction. (c) Size distribution of EB-NS exfoliated by bath sonication in different solvents and surfactants, measured by DLS. (d) Size distribution of EB-NS exfoliated by bath sonication in different solvents and surfactants. After sonication, the samples were divided by liquid cascade centrifugation. Larger particles are enriched in fraction 1, fraction 2 contains only particles *r*_hyd_ < 350 nm, fraction 3 contains only particles *r*_hyd_ < 175 nm. The samples were dispersed at constant volume and their fluorescence intensity was measured. Error bars: standard deviation, *N* = 1 independent sample, *n* = 5 technical replicates.

EB-NS were exfoliated after PB milling by both tip and bath sonication. Water was used as the exfoliation medium for both methods. DLS particle size analysis of EB-NS samples obtained by tip and bath sonication showed no significant differences, neither in the size distribution, nor in the size of the smallest particles obtained thereby (Fig. 1b). In both cases, the majority of particles had a size of 100–500 nm in radius and the smallest particles had a radius of 50 nm.

Tip sonication of EB for long periods at high energies is a relatively harsh method. All the energy required for the exfoliation process is transferred from the tip to the sample on a small contact area. At this point, the exposure to the tip is very high, so abrasion can occur there during sonication and material from the tip may be transferred into the EB-NS sample. After 6 hours of tip sonication with a previously freshly ground tip with a smooth surface, a clear abrasion and a change in the surface was visible (Fig. S2†). Particles abraded from the tip may contaminate the sample. Like other contaminating materials present in the sample, they are removed to a certain extent by the following centrifugation steps depending on their size and density. Only very small abraded particles with dimensions similar to the EB-NS would remain in the sample in this case. In the case of bath sonication, the energy is transferred to the sample *via* the water bath over the entire surface of the employed flasks, so that lower energy densities are likely to occur here and the risk of abrasion affecting the sample decreases.

In addition to the lower risk of impurities from the exfoliation process, bath sonication is able to exfoliate significantly higher amounts of EB-NS at the same time, as several bottles (>100 ml) of EB suspension can be placed in one bath sonicator and higher EB concentrations in the sonicated solution can be tolerated. On account of these two advantages, the following screening of surfactant media was carried out using bath sonication.

For the screening of exfoliation media, in addition to water, organic solvents and surfactants were investigated. The solvents propanol, dimethyl sulfoxide (DMSO), *N*-methyl-2-pyrrolidone (NMP), *N*-vinylpyrrolidone (VP) and *N*-cyclohexyl-2-pyrrolidone (CHP) have been successfully used for the exfoliation of other 2D materials such as graphene or tungsten and molybdenum sulfide.^14,24,26,51^ The surfactants sodium dodecyl sulfate (SDS) and sodium dodecyl benzyl sulfate (SDBS) were also found to be effective for exfoliation of 2D materials.^14,26,50^ They were both dissolved in deionized water, and one concentration below and one above the critical micelle concentration was tested for each surfactant. PB milled EB was dispersed in the respective solvent or surfactant solution at the same EB concentration of 2 g l^−1^.

Then DLS particle size analysis was performed on the EB-NS samples exfoliated by bath sonication in different media (Fig. 1c). Significant differences were found between the different solvents and surfactants, both for the size distribution of the nanosheets generated by exfoliation and for the size of the smallest nanosheets. DMSO, VP and CHP did not show significant differences compared to exfoliation in water. In all cases, the majority of particles were measured to be 100–500 nm in radius, with the smallest measured NS having a radius in the range of 50 nm. For exfoliation in propanol, both the average particle size and the minimum particle size were larger compared to all other solvents and surfactants. Therefore, it provides the poorest exfoliation performance of all media studied.

EB-NS exfoliated in the presence of surfactants exhibited the smallest average particle size and minimum particle size. The average hydrodynamic radius of these particles was reduced to hundreds of nm, the fraction of small particles with a radius <100 nm increased in comparison to water and organic solvents, and the lowest end of the size distribution was lowered, too. Among all tested samples, the one exfoliated in SDBS at a high concentration (17.5 g l^−1^) showed the highest yield of particles with a (hydrodynamic) radius <100 nm. It is important to note that DLS is not suitable to determine easily absolute sizes for non-spherical particles but it is able to pinpoint qualitatively to smaller or larger particles.

Additional to DLS particle size measurements, the EB-NS samples obtained from liquid phase exfoliation assisted by the various solvents and surfactants were divided into three different sized fractions by liquid phase centrifugation (Fig. 1d). All particles with a hydrodynamic radius *r*_hyd_ > 350 nm (and due to the methodology also a proportion of the smaller particles) were separated into fraction 1. Particles with *r*_hyd_ > 175 nm remained in fraction 2, while fraction 3 only contained particles with *r*_hyd_ < 175 nm. The EB-NS concentration in the different fractions were estimated from the fluorescence intensity. Similar to the DLS analysis, the solvents propanol, DMSO, NMP, CHP and VP were found to achieve a lower yield of small particles compared to sonication in water, whereas surfactant-assisted exfoliation lead to higher yields of smaller particles. With this method, the samples exfoliated in SDBS at high concentration (17.5 g l^−1^) also showed the highest yield of small particles.

Colloidal stability increased after PB milling and even more by sonication. All sonicated samples were colloidally stable for up to several days, with stabilities increasing with decreasing particle size. In summary, we identified a solvent (water) and a surfactant (SDBS) to obtain the highest yield and smallest size of EB-NS.

### Size and morphology of EB-NS

This exfoliation process (PB milling + surfactant assisted exfoliation by bath sonication) reduces the size of EB from large macroscopic particles to micrometer sized particles and down to EB-NS. Atomic force microscopy (AFM) measurements are able to provide more quantitative detail about the height and size of EB-NS.^28,30^ For this purpose, the exfoliation method and medium with the highest yield of small nanosheets (*r*_hyd_ < 175 nm) was selected for further investigation. EB-NS were prepared by PB milling and bath sonication in highly concentrated SDBS. The smallest fraction of nanosheets obtained by liquid phase centrifugation (*r*_hyd_ < 175 nm) was selected and spin-coated onto a mica surface for AFM investigation. Spin coating could affect the size distribution because larger EB-NS and EB-NS aggregates are more likely to be removed by centrifugal forces.

**Fig. 2:**
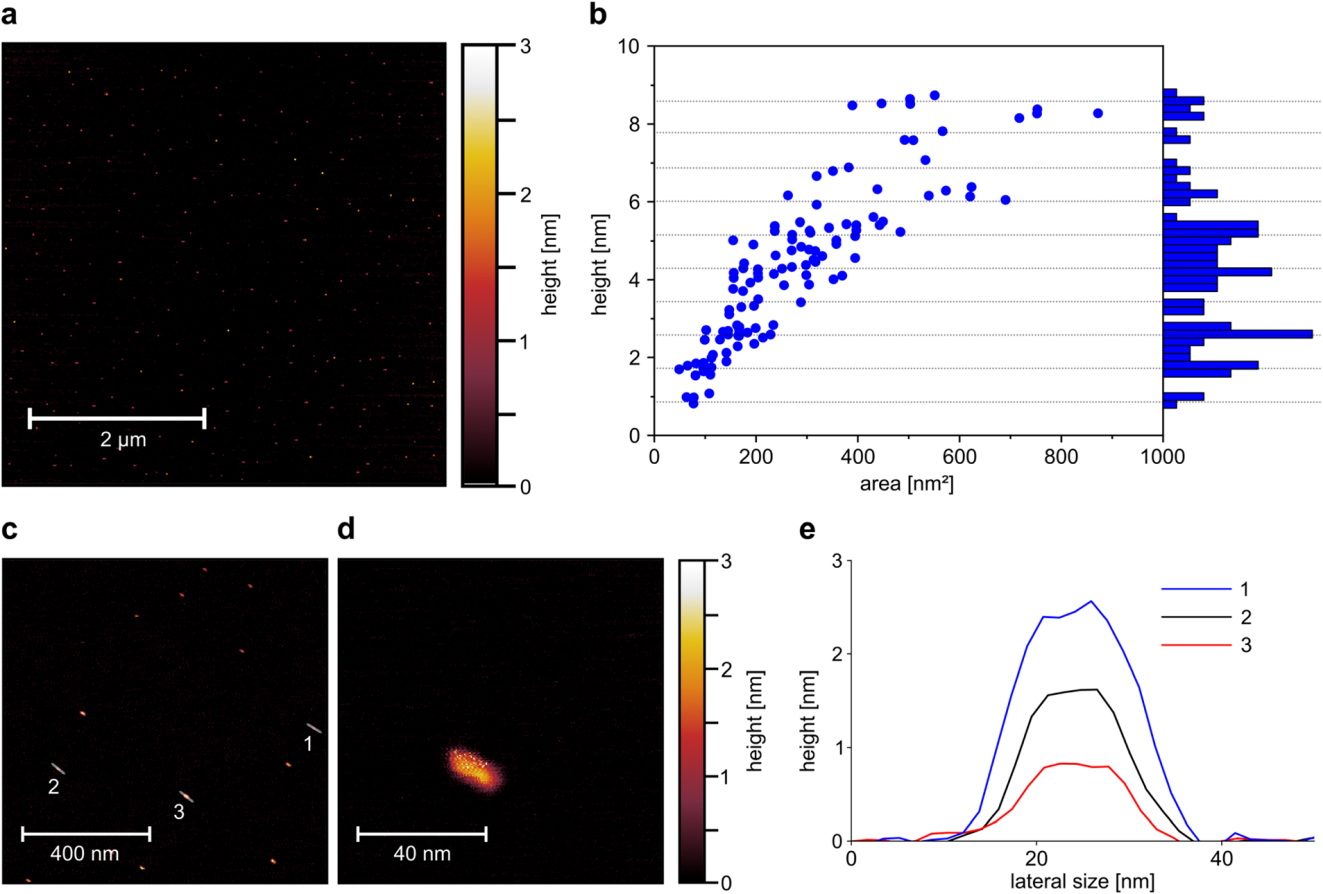
Height and morphology of exfoliated EB-NS (a) AFM overview image of EB-NS spin-coated on mica substrate shows the monodispersity of the sample on a larger scale. Most EB-NS have diameters <50 nm and heights <5 nm. (b) Surface area and maximum height distribution of *n* = 118 analyzed EB-NS. The histogram (*y*-axis) shows the height distribution for 0.2 nm bin sizes. Grey dotted lines show expected multilayer heights *h* = *n* × *d* (*n* = 1, 2,…) of NS with a monolayer height of *d* = 0.86 nm. (c) Magnified part of image (a) shows EB-NS in more detail. (d) Detailed image of one EB-NS. (e) Height profiles of three representative EB-NS marked in (c). The reported height profiles represent EB-NS with a height of one, two and three monolayers.

The increased monodispersity of this method can already be observed in low magnification AFM overview images (Fig. 2a). More than 100 EB-NS were observed on an area of several μm^2^. All particles had consistently small sizes with less than 5 nm in height, and lateral dimensions of less than 50 nm. No larger EB-NS were present in the sample, and agglomeration of the particles was negligible. In a magnified, more detailed AFM image, the shape of individual EB-NS became visible (Fig. 2c). All EB-NS had a height <3 nm and lateral dimensions in the range of 20 nm. All particles exhibited a similar shape and a slightly elongated profile. This shape is oriented in a similar direction for all particles, which is assumed to be due to the rotational forces during sample preparation by spin coating. The smallest observed height (≈0.9 nm) corresponds to the monolayer of EB bulk material reported in literature.^28,35,36^ A typical EB-NS showed a length of ∼20 nm, width of ∼10 nm and height of ∼2 nm (Fig. 2d and S3b†).

A constant aspect ratio between nanosheet height and diameter has been shown for EB-NS in previous work,^30^ as well as for other 2D-materials.^54^ For three of the EB-NS measured on the more detailed image, a height profile was generated along the longer axis of the nanosheets (Fig. 2e). These profiles show that, within the limits of measurement accuracy, the particles have a platelet shape with a constant height over most of their surface. The three measured EB-NS showed heights of 0.9 nm, 1.8 nm and 2.6 nm. The already known or predicted values for the height of EB monolayers indicate that these are EB-NS with one, two and three monolayers.^28,35^

To get more statistics, a larger number of EB-NS (Fig. 2b, c and S3a†) were analyzed. In these images, the maximum height and base area of the nanoparticles were assessed, thus allowing a calculation of the ratio of height to base area (Fig. S3c†). With this method, the relationship between height and lateral dimensions could further be clarified. The measured values correspond to those expected with a linear relationship between height and diameter, which results in the height increasing with the root of the base area (Fig. S3d†). In addition, there was no continuous height distribution, but the particles appeared to separate into groups of discrete height levels (Fig. 2b). We found heights of 0.9 nm equivalent to a single-layer nanosheet as well as heights between 1.5 and 1.8 nm, corresponding to two-layer nanosheets. Additionally, we found heights between 2.3 and 2.8 nm, corresponding to three-layer nanosheets.

The histogram of the height distribution showed maxima at multiples of the monolayer height which by this method is determined as 0.86 nm (Fig. 2b).

### Photophysical properties of EB-NS

**Fig. 3:**
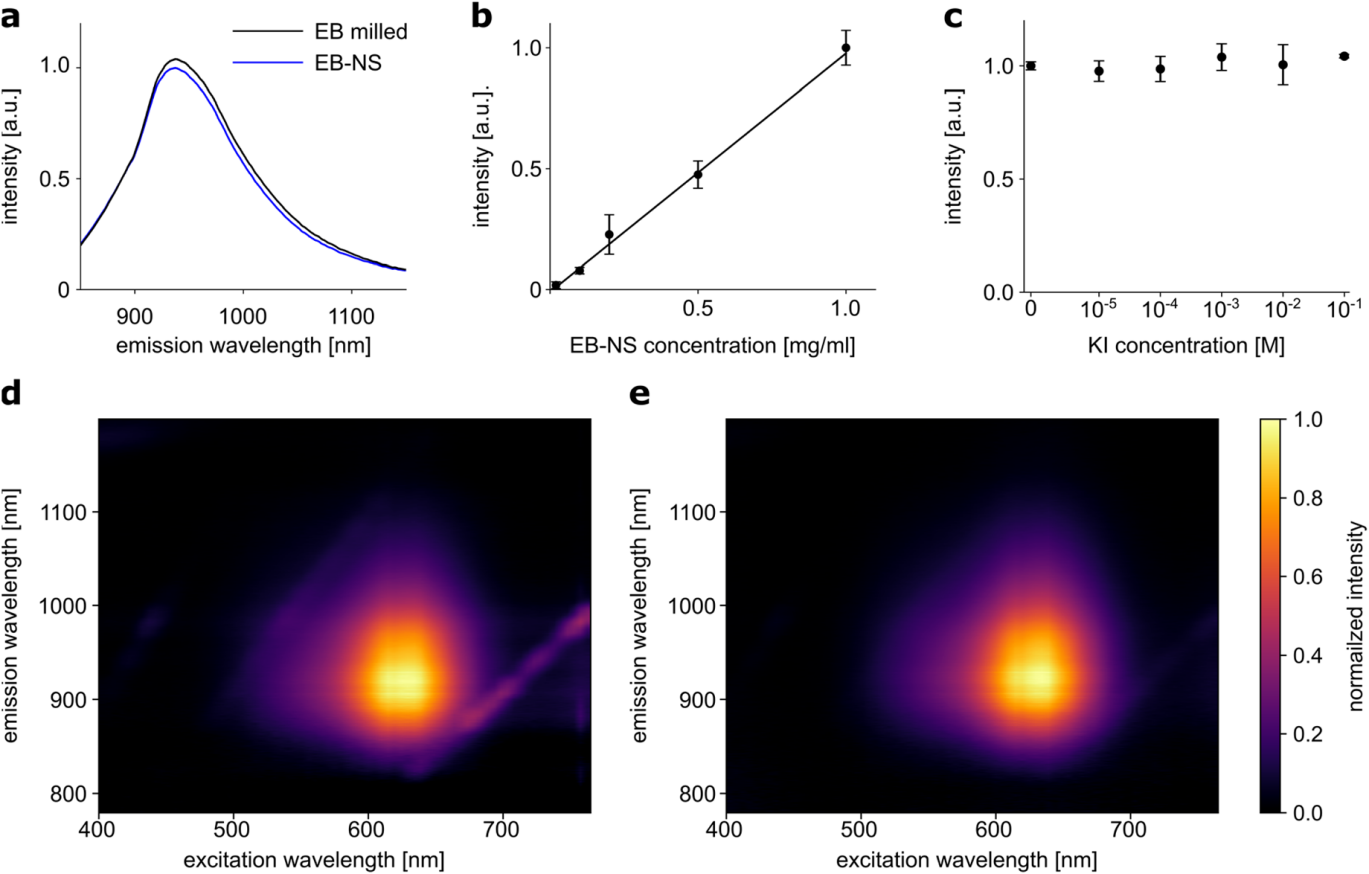
Photophysical properties of exfoliated EB-NS. (a) Emission spectrum of EB (milled, <10 μm particle size) and EB-NS (exfoliated by bath sonication in SDBS, fraction 3, *r*_hyd_ < 175 nm) upon laser excitation at 561 nm. Both samples are diluted to a concentration of 2 g l^−1^. (b) Fluorescence intensity of EB-NS increases linearly with concentration. Error bars: standard deviation, *n* = 3 samples. Black line: linear fit (*R*^2^ = 0.994). (c) Fluorescence intensity of EB-NS (exfoliated by bath sonication in SDBS, fraction 3, *r*_hyd_ < 175 nm) after adding increasing concentrations of a typical quencher (KI) to EB-NS. Error bars: standard deviation, *n* = 3 samples. (d) 2D excitation-emission spectrum of EB (milled, <10 μm particle size). (e) 2D excitation-emission spectrum of EB-NS (exfoliated by bath sonication in SDBS, fraction 3, *r*_hyd_ < 175 nm).

Fluorescence properties of a material may be altered or even lost during the transformation from bulk material to nanoparticles. For some fluorescent 1D materials, such as SWCNTs, it has been shown that the fluorescence quantum yield decreases with decreasing length.^55^ Such changes were not observed in the exfoliation of EB in water to large nanosheets.^30^ Following the assessment of particle sizes, we investigated whether the exfoliation of EB into nanosheets affected their fluorescence properties or the photostability of these nanomaterials could be confirmed. 1-D fluorescence spectra (laser excitation at 561 nm) showed no relevant differences between two EB or EB-NS samples for different steps of the exfoliation process, of comparable concentration (∼2 g l^−1^) but different particle sizes (Fig. 3a). Samples after milling and before sonication were compared with samples of the smallest size fraction (*r*_hyd_ < 175 nm) obtained from surfactant-assisted exfoliation. The intensity of the fluorescence remained high even for small particle sizes, and the peak of the maximum fluorescence emission did not change (935 nm). Furthermore, the fluorescence intensity increased in a linear fashion with concentration (Fig. 3b). This underlines that EB-NS are not easily affected by nearby other fluorophores.

2D excitation-emission spectra were also recorded from both samples with different particle sizes from different steps of the exfoliation process (Fig. 3d and e). Here, a very broad absorption in the green-red region of the spectrum was observed in both cases. Light with a wavelength between 550–760 nm could be used to excite both EB or EB-NS samples. Maximum excitation was observed at ∼630 nm, but due to the broadness of the spectrum, excitation with a laser at a wavelength of 561 nm yielded sufficiently strong fluorescence. Given that this source is ideal for excitation of SWCNTs as well, EB-NS and these fluorophores can be combined for advanced sensing concepts, *e.g.* multiplexed detection and ratiometric measurements.^45^ Similar to the excitation, the fluorescence emission did not change between EB or EB-NS of different particle sizes. For both samples, fluorescence is emitted in the range of 880–1000 nm with a maximum at 930–940 nm.

Some fluorophores are affected by the presence of other molecules. For example, the addition of potassium iodide (KI) to organic fluorophores leads to strong quenching of their fluorescence.^56^ The response to this analyte was investigated for EB-NS (Fig. 3c). EB-NS caused neither a change nor a shift in fluorescence emission in the presence of KI. Thus, the addition of KI at different concentrations to EB-NS had no significant effect on the NIR fluorescence. This suggests that EB-NS are very stable fluorophores independent of their chemical environment and the fluorescence is not due to surface states.

### Macroscopic patterning using EB-NS

The new exfoliation method is able to produce EB-NS in larger quantities, which is necessary for macroscopic applications. The benefits of bulk EB and its fluorescence has already been demonstrated. In addition to useful bioimaging applications,^30,40^ EB has been applied for fingerprint detection.^42,46^ Additionally, a first approach to print EB with a material printer has been demonstrated.^28^ Due to the bright fluorescence of EB-NS in the NIR, it should be possible to image them from a certain distance (stand-off imaging). For this purpose, we excited the fluorescence with a simple white light LED (equipped with a 700 nm short-pass filter to filter out NIR signals). The fluorescence was recorded with a simple and inexpensive CMOS camera (equipped with a 900 nm long-pass filter to detect only NIR signals).

**Fig. 4:**
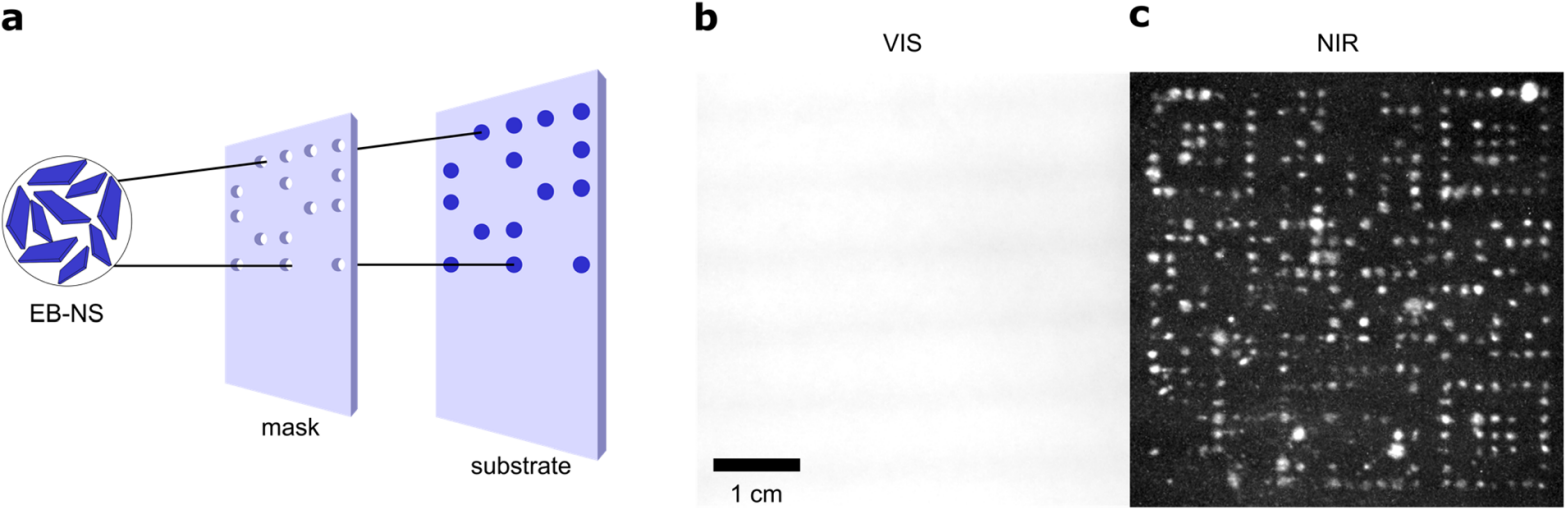
Macroscopic NIR patterning and barcoding using EB-NS. (a) Schematic of the barcoding process. A custom-made mask creates the desired pattern (*e.g.* a QR code). EB-NS dispersion (2 g l^−1^) is spray-painted though the mask on a paper substrate. (b) Image of the barcoded paper (in the visible). No pattern is visible. (c) NIR image (*λ* > 900 nm) of the paper shows the EB-NS pattern (excitation with white light LED and a 700 nm short-pass filter, image acquisition with CMOS camera and a 900 nm long-pass filter).

We tested EB-NS for NIR barcoding as a proof-of-principle experiment. In such applications, high brightness and availability in larger quantities are crucial. Our patterning approach used a simple home-made mask, which was created by simply cutting out specific shapes out of paperboard to be coated with EB-NS (Fig. 4a). This mask was placed over a paper substrate. EB-NS suspension (∼2 g l^−1^) was distributed over the mask using a standard spray bottle. This required a sufficient amount of EB-NS suspension in the ml range. The procedure allowed us to create the desired pattern of EB-NS a substrate such as paper.

With the low-cost optical setup described above, these patterns were imaged from distances >10 cm. Without a 900 nm long pass filter placed in front of the camera, the visible light of the LED is recorded by the camera (Fig. 4b). The strong reflection of the white excitation light overlaps with the fluorescence of the EB-NS and the patterns were not detected with the camera or the human eye. It is important to note that standard Si-based cameras have a much higher quantum yield for visible light than for NIR, which means that it is not expected to detect a NIR signal without filters.^57^ However, with the NIR long pass filter in front of the camera, only the NIR fluorescence emission was captured by the camera (Fig. 4c). Here, the EB-NS pattern was clearly visible. Further improvements of this process by more sophisticated printing techniques can be anticipated for the future.

## Conclusion

We have developed an improved method to exfoliate the calcium–copper–silicate Egyptian blue into NIR fluorescent nanosheets (EB-NS) by screening different surfactants and solvents. This results in a robust protocol consisting of ball milling, surfactant-assisted bath sonication with the surfactant SDBS in water and centrifugation. This procedure is milder and easier to handle. It provides access to EB-NS of small diameter and a higher level of monodispersity. EB-NS with heights down to one or a few monolayers can be produced. Moreover, it is also scalable to larger quantities. Furthermore, we show that the photophysical properties of EB-NS are not diminished and they represent an extremely stable fluorophore. Improvements in the preparation of EB-NS could become very important for applications that require environmentally save materials in larger quantities such as precision agriculture or biomedical imaging.^11,58–60^

In summary, our efficient large-scale exfoliation process makes applications such as the use of EB-NS as macroscopic NIR barcodes possible. It paves the way for further fundamental studies of the photophysical properties of EB-NS as well as applications in NIR photonics.

## Experimental

### Exfoliation of EB to EB-ES

EB powder was purchased from Kremer Pigmente, and EB-NS were prepared in a two-step exfoliation procedure: first, a planetary ball milling step was carried out using a Pulverisette 7 Premium Line (Fritsch) equipped with 20 ml ZrO_2_ beakers and 5 mm ZrO_2_ balls. 4 g of bulk EB powder were mixed in the beaker with 4 ml of deionized water to create a slurry. 80 5 mm ZrO_2_ balls were added to the beaker. The milling was performed at 900 rpm for 3 × 20 min with 5 min cooling break between each run. The resulting slurry was moved to a reaction tube and diluted with deionized water to a volume of 100 ml. Liquid phase centrifugation was used to remove particles with a hydrodynamic radius >1 μm afterwards. The centrifugation parameters were calculated from a modified Stokes equation^40,61^
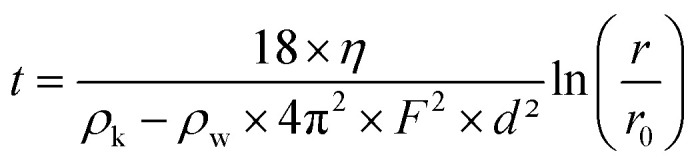
where *t* is the settling time, *η* the dynamic viscosity of the medium (water), *ρ*_k_ the grain density, *ρ*_w_ the medium density, *F* the rotation frequency, *D* the grain diameter, *r* the distance between the rotor's fulcrum and the sediment's height, *r*_0_ the distance between the rotor's fulcrum and the suspension's surface. All particles bigger than 1 μm settle down to the centrifugation pellet, the supernatant is decanted and stored. Water is added to the pellet, it is redispersed and the centrifugation step is repeated. Centrifugation, storage of the supernatant and redispersing of the pellet is repeated five times.

As second step, either tip or bath sonication was conducted. Tip sonication was conducted using a Fisherbrand Model 120 Sonic Dismembrator (Fisher Scientific). 50 ml EB-NS dispersion diluted with deionized water to 0.6 g l^−1^ were sonicated in an ice bath for 6 h at 72 W amplitude.

For the screening of different media in bath sonication, propanol, dimethyl sulfoxide (DMSO), *N*-methyl-2-pyrrolidon (NMP), *N*-vinylpyrrolidone (VP), *N*-cyclohexyl-2-pyrrolidone (CHP), sodium dodecyl sulfate (SDS) and sodium dodecyl benzyl sulfate (SDBS) were purchased from Sigma-Aldrich GmbH. Bath sonication was conducted using a Sonorex RK 103 ultrasonic bath (Bandelin Electronic). EB-NS were dispersed in the respective exfoliation media to a concentration of 2 g l^−1^. 15 ml of the so-obtained dispersions were filled in a 25 ml glass bottle and sealed using PTFE tape. The glass bottle was placed in the bath sonicator for 6 h at 60 °C. To compensate for irregularities in the energy distribution within the ultrasonic bath, three bottles were always evenly distributed in the ultrasonic bath at the same time and their contents were mixed after sonication. For further analysis, the particles were dried and dispersed in ultrapure water in another short sonication step in order to ensure comparable measurements.

### DLS particle size measurements

DLS particle size measurements were performed using a DynaPro NanoStar (Wyatt Technology) DLS instrument. EB-NS were suspended in deionized water to a concentration of 1 g^−l^ and 30 μl of the so-obtained dispersions were measured in a JC-551 Quartz Microcuvette (Wyatt Technology). The working temperature was fixed to 25 °C. A preinstalled measurement routine consisting of ten measurements was used to obtain the average particle size distribution and calculate the standard deviation.

### Liquid cascade centrifugation

Following the sonication step, the sample was divided in different size fractions using liquid cascade centrifugation. Three centrifugation steps were conducted with increasing centrifugation frequencies. Three fractions were aimed: particles with a hydrodynamic radius <350 nm, <175 nm and one fraction containing the remaining larger particles. Centrifugation conditions were calculated using the modified Stokes equation. After each centrifugation steps, the centrifugation pellet containing particles with larger hydrodynamic radius is stored. The supernatant contains particles with lower hydrodynamic radius. It is decanted and undergoes the next centrifugation step to further sort the particles by their size.

### NIR fluorescence spectroscopy

For NIR fluorescence spectroscopy a custom-made setup was used containing two light sources. For 1D-spectra, a Gem 561 laser (Novanta) was employed, for 2D imaging a MSH-150 monochromator (LOT-Quantum Design) equipped with a xenon arc lamp and a diffraction grating was used. Both light sources were connected to a IX73 microscope (Olympus) equipped with a 20× objective. For spectroscopy, a Shamrock 193i spectrograph (Andor Technology) coupled to an array NIR detector (Andor iDUs InGaAs 491) was connected to the microscope. For spectroscopy measurements, 200 μl EB-NS were introduced in a 96-well plate positioned above the objective. Fluorescence data was acquired *via* the Andor SOLIS software. For 1D fluorescence spectra, the laser power was set to 100 mW, exposure time to 1 s and input slit width to 500 nm. For 2D fluorescence spectra, the monochromator light was scanned in steps of 5 nm over the wavelength range 400–750 nm, for every measurement, the exposure time was set to 10 s and input slit width to 500 nm. Data analysis and plotting were performed using Python. For 2D measurements, correction for the quantum efficiency of the detector and for the spectral irradiance of the xenon lamp were performed.

### Atomic force microscopy

For atomic force microscopy, Egyptian blue nanoparticles were milled, and exfoliated *via* bath sonication in SDBS solution. By liquid phase centrifugation, particles with a hydrodynamic radius >175 nm were removed from the solution. The concentration was set to ∼1 mg ml^−1^. The samples were prepared by spin-coating the so-obtained particle suspension. 20 μl of the so-obtained suspension were spin-coated on mica substrate using a WS-400 lite spin processor (Laurell Technologies). The substrate was kept spinning on 1000 rpm for 2 min.

A Bruker BioScope Resolve AFM was employed in PeakForce Quantitative Nanomechanical Mapping (PF-QNM) mode in ambient environment (air) at 2 kHz resonant frequency. A Bruker ScanAsyst-Air probe was used (spring constant 0.4 Nm^−1^, resonance frequency 70 kHz, triangular geometry, nominal radius 2 nm) utilizing a scan rate of 1 Hz (resolution of 256 × 256) and a peak force setpoint of 1.8 nN with a peak force amplitude of 140 nm. Image analysis was performed with Gwyddion 2.61 software.

### Visible/NIR imagining

Visible and NIR images of patterned substrates were acquired using a white light source (UHP, Prizmatix) for excitation, equipped with a 700 nm (FESH0700, Thorlabs) and a 750 nm (FESH0750, Thorlabs) short-pass filter mounted in series to exclude NIR emission from the light source itself. Images were acquired with a DCC3240M camera (Thorlabs). For NIR images, it was equipped with a 900 nm (FEL0900, Thorlabs) long-pass filter, which was dismounted for visible images. Exposure times between 10 ms (visible) and 100 ms (NIR) were used for image acquisition.

## Author contributions

SK and BH conceived and designed the study. SK coordinated the study. BH and SA worked on various exfoliation methods and studied the EB-NS size distributions with input from GS. BH, AA and KT collected and analyzed the AFM dataset. BH measured 1D and 2D excitation and fluorescence spectra and performed the macroscopic patterning. BH and SK wrote the manuscript, with contributions from all authors.

## Conflicts of interest

There are no conflicts to declare.

## Supplementary Material

RA-013-D3RA04083F-s001
