# Supplementary material for: Surfactant assisted exfoliation of near infrared fluorescent silicate nanosheets
Source: RSC Adv. 2023 Jul 11;13(30):20916–25. doi: 10.1039/d3ra04083f (PMC10334366; doi:10.1039/d3ra04083f)
Supplement: RA-013-D3RA04083F-s001 [file RA-013-D3RA04083F-s001.pdf]

Electronic Supplementary Material (ESI) for RSC Advances.
This journal is © The Royal Society of Chemistry 2023

Supplementary Information

Surfactant Assisted Exfoliation of Near Infrared Fluorescent Silicate Nanosheets

Björn Hill,[a] Smitha Abraham,[a] Anas Akhtar,[b] Gabriele Selvaggio,[a] Kristina Tschulik[b] and Sebastian Kruss[*acd]

[a.] *Department of Chemistry, Ruhr Universität Bochum, 44801 Bochum, Germany E-mail: Sebastian.Kruss@rub.de*
[b.] *Analytical Chemistry II, Ruhr Universität Bochum, 44801 Bochum, Germany*
[c.] *Fraunhofer Institute for Microelectronic Circuits and Systems, 47057 Duisburg, Germany*
[d.] *Center for Nanointegration Duisburg-Essen (CENIDE), 47057 Duisburg, Germany*

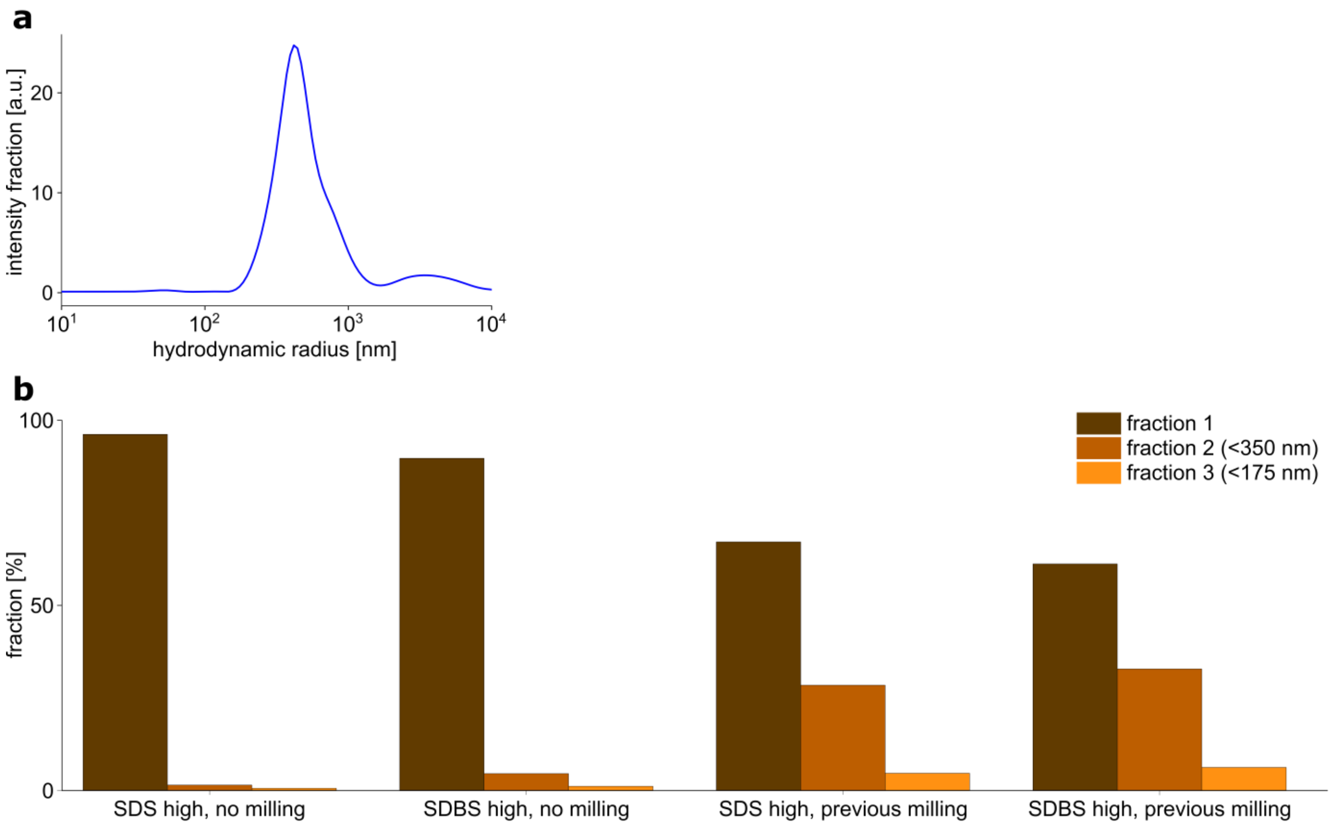

Figure S1: Liquid phase exfoliation of EB-NS a) Size distribution of EB-NS after planetary ball milling and size-selective centrifugation, measured by DLS. b) Size distribution of EB-NS exfoliated with and without previous planetary ball-milling exfoliated by bath sonication in different surfactants. After sonication, the samples were divided by liquid cascade centrifugation. Larger particles are enriched in fraction 1, fraction 2 contains only particles r_{hyd}<350 nm, fraction 3 contains only particles r_{hyd}<175 nm. The samples were dispersed at constant volume and their fluorescence intensity was measured.

a b

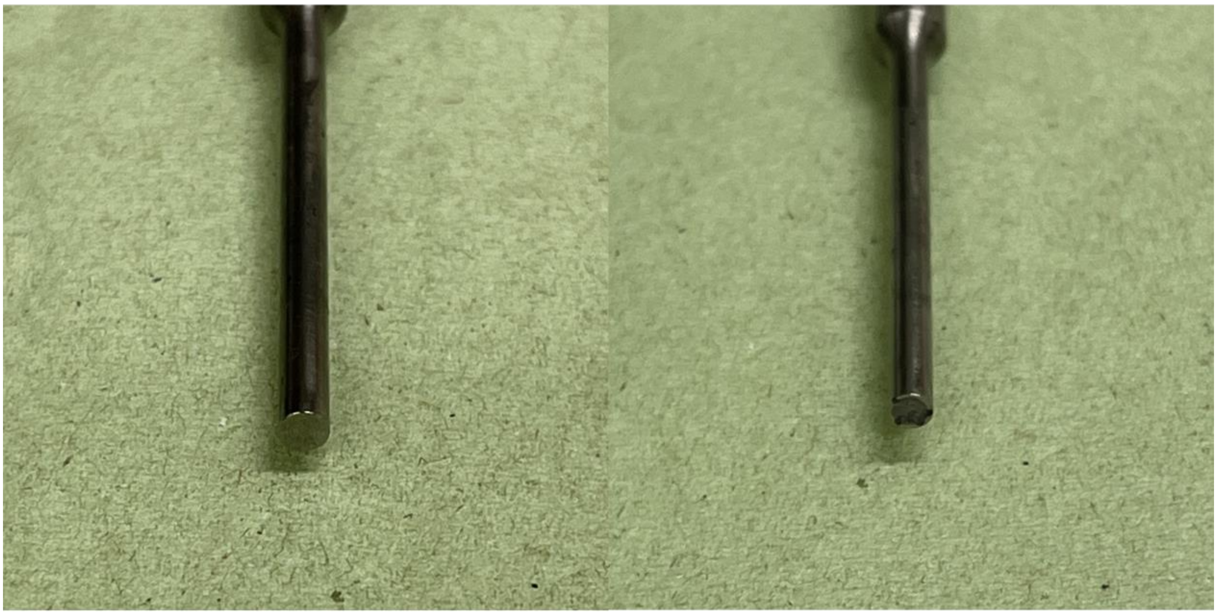

Figure S2: Tip erosion upon tip sonication of EB a) Photograph of a sonication tip for a sonic dismembrator. The tip is freshly ground and has a flat, smooth surface. b) Photograph of the same sonication tip for a sonic dismembrator after sonicating EB dispersion. 50 ml EB-NS dispersion diluted with deionized water to 0.6 mg ml^{-1} were sonicated in an ice bath for 6 h at 72 W amplitude. The tip shows abrasions, it has small cavities in the surface. The weight of the tip has decreased by 4 mg after sonication.

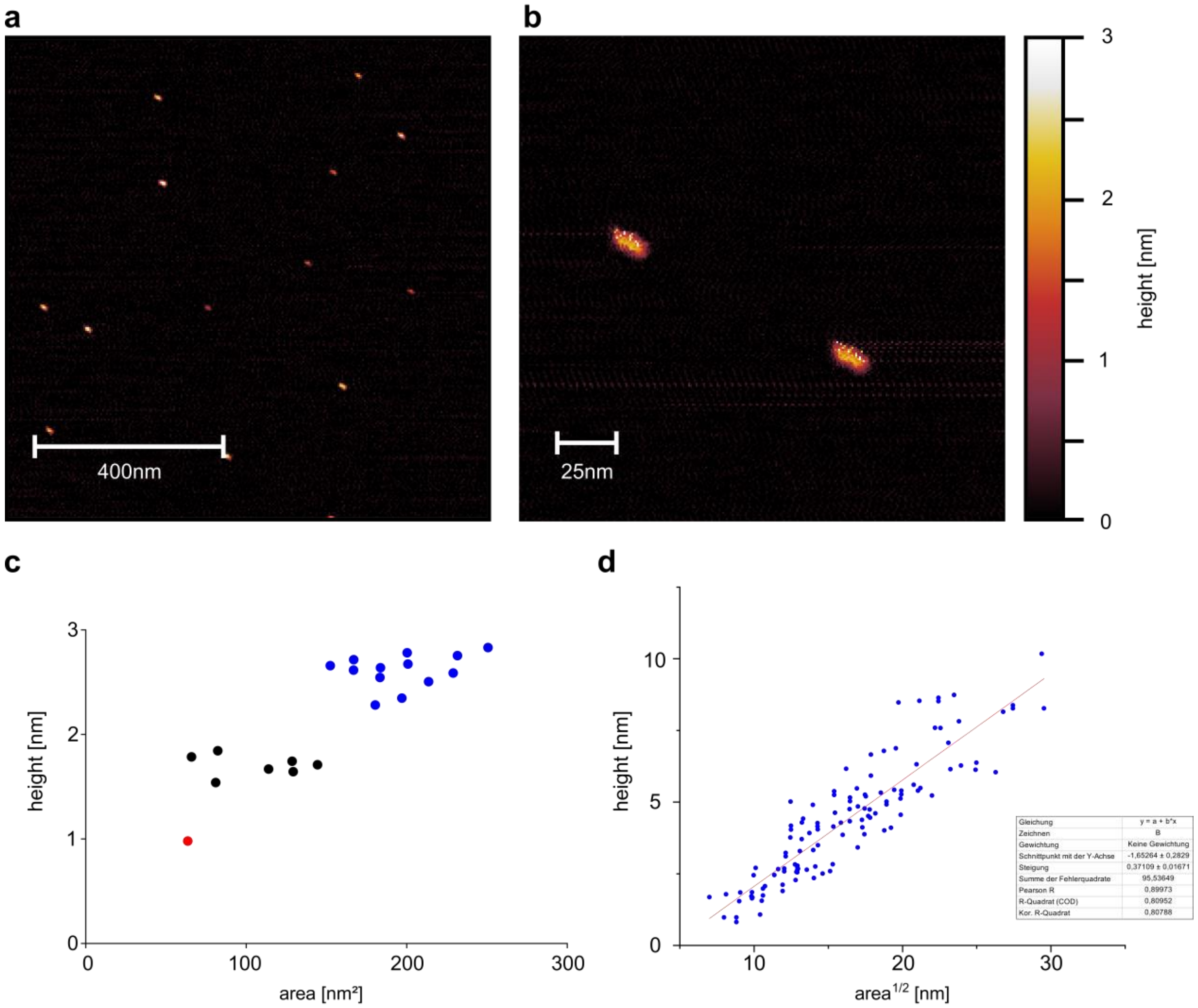

Figure S3: Height and morphology of exfoliated EB-NS a) Zoom in Figure 2a) shows the EB-NS in more detail. EB-NS are shaped slightly elongated. b) Detailed image of two EB-NS. c) Surface area and maximum height distribution of n=21 analyzed EB-NS from a) and Figure 2a). The different colors show particles expected to consists of 1,2 and 3 monolayers. c) Zoom in image a) shows the EB-NS in more detail. EB-NS are shaped slightly elongated. d) Surface area and maximum height distribution of n=118 analyzed EB-NS from Figure 2b). On the x-axis, the root of the base area is given. Red line: linear fit.
